# Therapeutic Potential and Mechanisms of Bee Venom Therapy: A Comprehensive Review of Apitoxin Applications and Safety Enhancement Strategies

**DOI:** 10.3390/ph17091211

**Published:** 2024-09-14

**Authors:** Maksymilian Stela, Natalia Cichon, Aleksandra Spławska, Monika Szyposzynska, Michal Bijak

**Affiliations:** 1 Biohazard Prevention Centre, Faculty of Biology and Environmental Protection, University of Lodz, Pomorska 141/143, 90-236 Lodz, Poland; maksymilian.stela@biol.uni.lodz.pl (M.S.); michal.bijak@biol.uni.lodz.pl (M.B.); 2 CBRN Reconnaissance and Decontamination Department, Military Institute of Chemistry and Radiometry, Antoniego Chrusciela “Montera” 105, 00-910 Warsaw, Poland

**Keywords:** bee venom therapy, melittin, apamin, anti-inflammatory therapy, anticancer therapy, natural compounds, allergy

## Abstract

Apitoxin therapy (BVT—bee venom therapy) is an emerging complementary treatment utilizing bee venom for various medical conditions. This review explores the potential and therapeutic mechanisms of bee venom, focusing on its chemical composition and the methods for its extraction and purification to enhance safety while maintaining bioactivity. Bee venom contains amphipathic peptides such as melittin and apamin, enzymes like phospholipase A2, and bioamines including histamine and catecholamines, contributing to its pleiotropic effects. The therapeutic applications of bee venom span anti-inflammatory, analgesic, antimicrobial, antiviral, neuroprotective, anti-arthritic, and anti-cancer activities. Clinical and laboratory studies have demonstrated its efficacy in treating chronic and autoimmune diseases, pain management, and improving quality of life. The immunogenic properties of bee venom necessitate ongoing research to mitigate allergic reactions, ensuring its safe and effective use in medical practice. This review summarizes the current state of research on bee venom therapy, highlighting its potential benefits and future research directions.

## 1. Introduction

Therapies based on natural compounds can be effective complements to conventional treatments for many diseases. Advances in the purification, isolation, and synthesis of natural compounds are intensifying scientific research into their therapeutic efficacy. One such therapy is apitoxin therapy (BVT, bee venom therapy), which utilizes bee venom in the treatment of various ailments [1]. The venom of the honeybee (*Apis mellifera*) contains a rich array of pharmacologically active compounds that exert pleiotropic effects on the human body.

One variation is apipuncture, which involves using bee venom to stimulate acupuncture points on the patient’s body. It merges traditional acupuncture methods with the medicinal benefits of bee venom. The venom is directly administered to specific acupuncture points chosen based on traditional Chinese medicine or other acupuncture-based medical systems. Once bee venom is applied to these points, they are stimulated to influence the body’s energetic flow (chi) and physiological functions [2]. In contrast, live bee sting therapy involves deliberately introducing bee venom into the body via a live bee’s sting. During the procedure, a live bee is positioned on the patient’s skin to provoke a sting. The venom is then injected into the skin, initiating its effects almost immediately. This therapy necessitates careful supervision by a qualified specialist who can manage potential allergic reactions and ensure patient safety, given the inherent risks involved. Despite these considerations, live bee sting therapy is increasingly favored as an alternative to conventional treatments [3]. Bee venom injections involve the direct administration of bee venom to a patient using a needle. This therapeutic procedure is performed by a qualified therapist who precisely determines the location and depth for injecting the venom. The dosage and frequency of injections are tailored to the specific requirements of the patient and the nature of the disease being treated. Bee venom injections represent a well-defined and closely monitored therapeutic approach related to the use of bee venom [4]. Topical application of bee venom involves directly applying bee venom to the skin surface. This method harnesses the therapeutic properties of bee venom without the need for injections. Bee venom can be administered in the form of creams, ointments, or gels, directly targeting the affected area of the skin. This approach offers a less invasive alternative to injections, catering to individuals who prefer to avoid needles or in cases where systemic absorption is unnecessary [5]. Topical application of bee venom is known to alleviate pain and inflammation and promote the healing of various skin conditions. While generally considered safe, it is important to administer bee venom topically under the supervision of a qualified healthcare professional. This ensures careful monitoring for any potential adverse reactions, particularly in individuals with a known allergy to bee products [6]. Bee venom electrotherapy involves integrating the therapeutic benefits of bee venom with electrical stimulation to enhance its efficacy in treating various medical conditions. Similar to traditional methods of bee venom therapy, bee venom is either applied topically to the skin or injected into specific locations on the body. However, in bee venom electrotherapy, electrodes are also positioned on the skin near the injection site [7]. Once in place, a low-intensity electric current is delivered through these electrodes. The primary objective of this electrical stimulation is to facilitate the absorption of bee venom into the tissues, promote circulation, and potentially amplify the therapeutic effects of the bioactive compounds found in the venom. This combined approach is believed to synergistically enhance treatment outcomes by improving local blood flow, reducing inflammation, modulating pain perception, and potentially expediting tissue repair processes [8]. The characterization of bee venom therapy methods is presented in Table 1.

Chemical analysis capabilities have enabled the identification of its chemical composition, which includes amphipathic polycationic peptides such as melittin and apamin, enzymes such as phospholipase A2, and low-molecular-weight compounds including active biogenic amines like histamine and catecholamines [10]. The diversity of chemical components in bee venom has sparked interest and collaboration within the scientific and clinical communities [11,12].

Recently, there has been a growing interest in the therapeutic use of bee venom. Doctors and licensed apitherapists are using bee venom to treat patients suffering from chronic and autoimmune diseases. Both contemporary clinical studies and laboratory tests confirm that bee venom is an excellent form of biotherapy, demonstrating a variety of properties, including antimutagenic, antinociceptive, radioprotective, antihepatotoxic, cytoprotective, antioxidant, antimicrobial, antiviral, anti-inflammatory, neuroprotective, antiarthritic, antimetastatic, and anticancer effects [7,9,13,14,15]. Bee venom can help combat inflammation and inhibit the degradation of connective tissue, as well as restore activity and mobility, supporting the body’s natural defenses. Furthermore, in addition to its therapeutic effects, literature reports suggest that bee venom may reduce the side effects of conventional medications [16]. The therapeutic use of bee venom is generally regarded as relatively safe. However, it comprises several potent allergenic compounds, including melittin, phospholipase A2, and hyaluronidase. In most cases, a bee sting induces a mild physiological response, typically involving localized pain and minor dermal edema. Nonetheless, a subset of individuals may experience a severe hypersensitivity reaction, potentially culminating in significant inflammation and anaphylactic shock [17]. The abundance of biologically active substances found in bee venom has stimulated scientific interest and collaborative efforts between researchers and clinicians. These discoveries open new avenues for the development of therapies based on natural compounds, which may serve as effective adjuncts to conventional treatments.

Due to its strong immunogenic effects, efforts are currently being made to remove the most allergenic proteins. Therefore, this review attempts to describe the methods of obtaining and purifying bee venom that preserve its bioactive properties while significantly enhancing the safety profile of the therapy.

## 2. Application of Bee Venom in Therapy

### 2.1. Analgesic and Anti-Inflammatory Activity of Bee Venom

The effectiveness of bee venom therapy primarily stems from its analgesic and anti-inflammatory properties. Bee venom is commonly used to alleviate pain, primarily through injections at acupuncture points, such as ST36 (Zusanli) [18]. The application of bee venom alleviated visceral pain in rodents administered intraperitoneal acetic acid, with greater analgesic efficacy observed when bee venom was applied to acupuncture points compared to random sites on the body [19].

In recent years, bee venom and its main components, including melittin and phospholipase A2, have been reported to alleviate pain of various origins, such as neuropathic pain associated with degenerative joint disease, burns, etc. [15,20,21,22,23,24]. It is suggested that the antinociceptive action of bee venom may be mediated through α2-adrenergic receptors. In a study where inflammation was induced in rats by collagen, administration of an α2-adrenergic receptor antagonist (yohimbine) blocked the antinociceptive effect of bee venom, in contrast to naloxone (another an opioid receptor antagonist) [25]. This confirms the involvement of α2-adrenergic receptors in the analgesic effect of bee venom.

Further supporting this, studies by Li et al. showed that concurrent therapy with bee venom and venlafaxine induced a lasting and additive analgesic effect on mechanical allodynia induced by paclitaxel-induced cold allodynia and mechanical allodynia in mice [21]. Additionally, bee venom acupuncture inhibited excessive neuronal activation in WDR neurons in the spinal cord caused by cold and mechanical allodynia in rats treated with vincristine, involving the descending noradrenergic pathway [18]. The antinociceptive effect of bee venom acupuncture has also been confirmed in the treatment of neuropathic pain in animal models. For instance, experiments with rats subjected to chronic constriction injury of the sciatic nerve indicated that α2-adrenergic receptors are responsible for the pain-relieving response to bee venom acupuncture, whereas opioid receptors do not influence this process, as shown by administering opioid receptor inhibitor (naloxone) and α2-adrenergic receptor inhibitor (idazoxan) to the animals. Therefore, this proposed therapy may be an effective alternative for patients with painful peripheral neuropathy, particularly those who respond inadequately to opioid analgesics [26]. Moreover, Kim et al. demonstrated that formalin-induced pain behaviors were alleviated, associated with inhibition of Fos transcription factor expression in the spinal cord [27].

**Figure 1:**
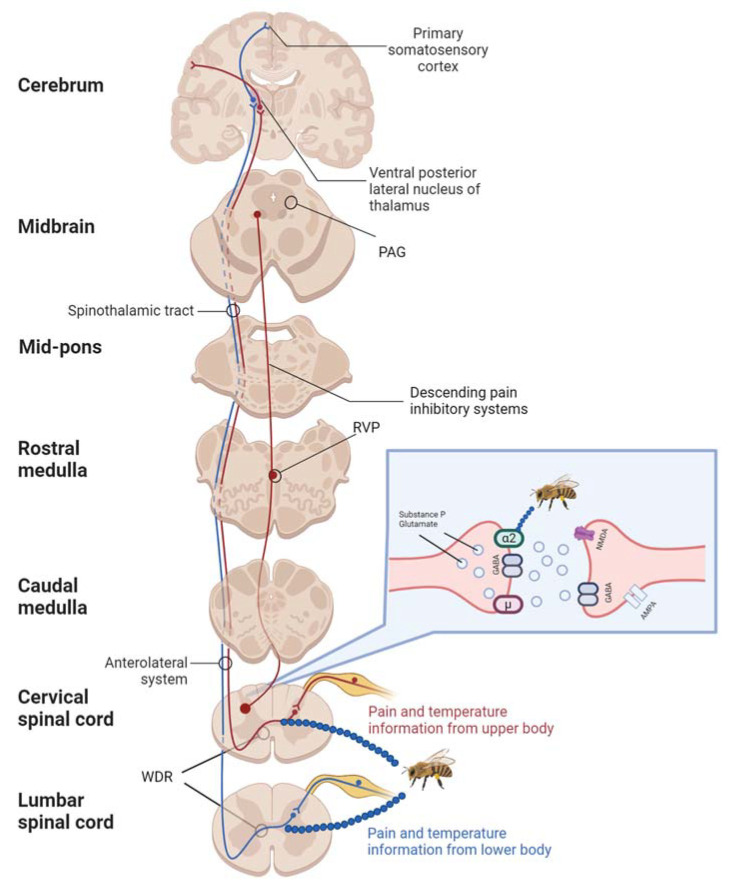
Antinociceptive mechanisms of bee venom: Bee venom exerts its antipain effects by activating descending pain inhibitory systems, which involve pathways from the rostral ventromedial medulla (RVM) to the spinal cord. Additionally, it activates presynaptic α2-adrenergic receptors in the dorsal horns of the spinal cord, leading to reduced release of the pain-related neuropeptide substance P into the synaptic cleft. The blue dots indicate the specific sites of action for the bee venom. Created in BioRender. Bijak, M. (2024) BioRender.com/o01c588 (accessed on 13th Sep 2024).

Descending pain inhibitory systems (DPISs), which originate in the midbrain and medulla oblongata, play a crucial role in modulating or inhibiting pain transmission. Key neurotransmitters involved in the descending pathway include serotonin and noradrenaline (Figure 1). Many analgesic drugs, such as tramadol, act directly on this pathway by inhibiting the reuptake of serotonin at synapses, thereby enhancing serotoninergic transmission. DPISs are controlled by cells regulated by opioidergic and GABAergic systems [28]. The mechanism of action of opioid analgesics (such as fentanyl and morphine) involves crossing the blood–brain barrier and activating the descending pathway through stimulation of opioid receptors in the midbrain [29]. Similarly, melittin exerts its analgesic effects through DPISs, as demonstrated in rodent models [30]. Subsequent studies have shown that administering methysergide, a serotonin receptor agonist, abolished the analgesic effects of bee venom, while opioid receptor antagonists like naltrexone and naloxone did not effectively reverse antinociception (Figure 1) [19,25,26,30].

In recent randomized studies, such as that by Seo et al., bee venom acupuncture has been shown to contribute to alleviating pain symptoms and thereby improving quality of life. In this study, 54 patients aged 18–65 with chronic low back pain lasting at least three months participated. Participants were divided into respective study groups receiving either acupuncture with bee venom or placebo using the same treatment protocol [31]. Similarly, in another study involving 40 patients with knee osteoarthritis, bee venom acupuncture administered twice weekly for four weeks demonstrated significant pain relief compared to 20 individuals treated with conventional acupuncture. Injection of bee venom alleviates pain symptoms by inhibiting inflammatory processes in joints, likely through reducing lipopolysaccharide (LPS)-induced production of prostaglandin E2 (PGE2) via inactivation of NF-κB dependent on the JNK kinase pathway [32,33,34,35]. Bee venom, through TNF-α activity, can stimulate the activation of nuclear factor kappa light chain enhancer of activated B cells (NF-κB) and inhibit the expression of genes encoding cyclooxygenase 2 (COX-2), inducible nitric oxide synthase (iNOS), cytosolic phospholipase A2 (cPLA2), and interleukin 1β (IL-1β) [36].

**Figure 2:**
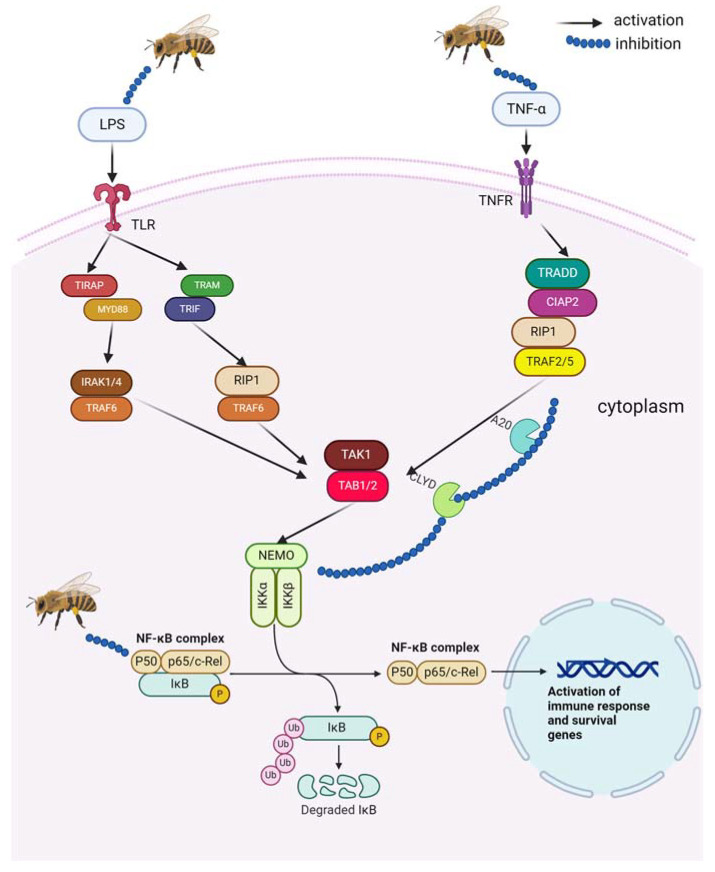
Mechanism of anti-inflammatory action of bee venom: Bee venom modulates TNF-α activity, leading to the downregulation of pro-inflammatory genes such as *COX-2*, *iNOS*, *cPLA2*, and *IL-1β*. This effect is mediated by inhibiting NF-κB binding to DNA through stabilizing IκBα and preventing p50 translocation to the nucleus. Additionally, bee venom may inhibit the production of LPS-induced PGE2 Created in BioRender. Bijak, M. (2024) BioRender.com/u38o134 (accessed on 13th Sep 2024).

Inhibition of iNOS and COX-2 gene expression results from blocking NF-κB binding in the promoter region of these genes, achieved through interaction with IκBα (nuclear factor of kappa light polypeptide gene enhancer in B-cell inhibitor, alpha). IκBα protein inactivates NF-κB transcription factor by masking nuclear localization signals and maintaining it in an inactive state in the cytoplasm. NF-κB typically forms a dimer most commonly composed of p50 and RelA proteins. IκBα can bind and block NF-κB activity only when unphosphorylated. Conversely, phosphorylation of inhibitory protein IκBα by IKK (IκB kinase) leads to dissociation of IκBα from NF-κB. Freed NF-κB translocates to the nucleus and activates the expression of various genes, including those encoding pro-inflammatory proteins. Bee venom may also prevent NF-κB from binding to DNA by inhibiting the translocation of p50 protein to the cell nucleus (Figure 2) [32,33,34,35]. In vitro studies on the human chondrosarcoma cell line HTB-94 exposed to LPS demonstrated that bee venom administration reduced the expression of genes involved in the inflammatory process, including interleukin 6 receptor (IL-6), matrix metalloproteinase 15 (MMP-15), caspase 6, and tissue inhibitor of metalloproteinase-1 (TIMP-1) [34]. Furthermore, the anti-inflammatory action of bee venom was associated with decreased production of NO and TNF-α, which contribute to the influx of inflammatory cells and consequent joint erosion and cartilage and bone destruction observed in arthritis [37,38].

Importantly, bee venom application also alleviated pain in patients with central pain syndrome occurring 60 days post ischemic stroke. Eight patients were divided into two experimental groups, where one group received acupuncture point injections containing diluted bee venom (0.005% in saline) (injection points included acupuncture points LI15, GB21, LI11, GB31, ST36, and GB39 on the affected side), while the other group received saline injections only. Injections were administered twice a week for three weeks. The experimental group showed significant improvement in subjective perception of central post-stroke pain compared to the control group [39].

### 2.2. Rheumatoid Arthritis

Rheumatoid arthritis (RA) is a severe autoimmune disease affecting approximately 0.5% of the population. RA results in irreversible joint and bone destruction, causing joint pain, severe disability, and even premature death [40]. In addition to conventional pharmacotherapy and rehabilitation, various forms of alternative therapy are used for RA, including bee venom therapy. Chang and Beliven were the first to document that subcutaneous injection of bee venom could reduce paw edema in rats and adjuvant-induced arthritis [41]. Furthermore, clinical studies have shown that bee venom administration to RA patients reduces pain, swelling, and morning stiffness of affected joints [42]. The alleviation of RA symptoms is likely due to bee venom’s ability to inhibit lipopolysaccharide (LPS)-induced reactive oxygen species (ROS) production and intracellular calcium release. Therefore, the anti-arthritic effect of bee venom may be associated with its ability to suppress the production of pro-inflammatory mediators (PGE2, NO, TNF-α), as well as ROS and calcium release via activation of the JNK pathway discussed above [43].

Hong et al. observed that rheumatoid synovial cells exposed to bee venom exhibited characteristics of apoptotic cells [44]. Furthermore, the induction of apoptosis was associated with reduced Bcl-2 expression and increased levels of Bax protein and caspase 3. Additionally, bee venom may enhance the efficacy of other drugs while simultaneously reducing their adverse effects. Darwish et al. demonstrated that bee venom intensified the anti-arthritic effects of methotrexate, while limiting its hepatotoxicity [45]. Similarly, Liu et al. observed that combined therapy with bee venom and other drugs used in RA patients was more effective than monotherapy. Importantly, their study showed that the use of bee venom allowed for a reduction in the dosage of anti-rheumatic drugs and decreased the frequency of relapses [46].

Bee venom extracted from *Apis mellifera* under the trade name Apitox^®^ (Apimeds, Inc., Seongnam-si, Republic of Korea) has been clinically approved in the Republic of Korea for the treatment of osteoarthritis based on phase III clinical trial results involving 363 patients with knee osteoarthritis [47]. Furthermore, the U.S. Food and Drug Administration (FDA) has approved clinical trials on the use of Apitox^®^ for alleviating pain and swelling associated with rheumatoid arthritis, tendonitis, bursitis, and multiple sclerosis [48]. Additionally, Apimeds sponsors clinical studies on the use of Apitox^®^ for treating other conditions [47].

### 2.3. Neurodegenerative Diseases

#### 2.3.1. Parkinson’s Disease

Parkinson’s disease (PD) is a neurodegenerative disorder characterized by progressive degeneration of dopaminergic neurons in the substantia nigra pars compacta, striatum, and cortex. This neuronal loss results in dopamine deficiency, accompanied by excessive activity of glutamatergic neurons that inhibit the activity of the thalamus [49]. It is estimated that 1–2% of the population over the age of 60 suffers from PD. Symptoms of PD worsen with disease duration, initially involving slowed movement and writing difficulties, followed by bradykinesia, muscle rigidity, resting tremor, postural instability, and reduced speech volume and clarity [50].

Previous studies suggest the therapeutic potential of bee venom components in treating central nervous system disorders, including PD [51,52]. In a mouse model of PD, bee venom therapy prevented the degradation of dopaminergic neurons in the substantia nigra and striatum, and increased dopamine levels [53]. Another study demonstrated that bee venom protected dopaminergic neurons in the substantia nigra from the neurotoxic effects of 1-methyl-4-phenyl-1,2,3,6-tetrahydropyridine (MPTP), which induces PD development. Cellular analysis indicated that acupuncture with bee venom prevented the MPTP-induced loss of tyrosine hydroxylase immunoreactivity in the substantia nigra and striatum. Moreover, this form of acupuncture attenuated the MPTP-induced phosphorylated c-Jun immunoreactivity in the substantia nigra [51]. Additionally, bee venom reduced microglial cell activity and inhibited the infiltration of CD4+ cells, including helper T lymphocytes, monocytes, macrophages, and dendritic cells [54]. Khalil et al. demonstrated in a mouse model of PD that bee venom administration improved motor abilities while reducing dopamine, norepinephrine, glutathione, and serotonin levels. Bee venom administration also lowered levels of pro-inflammatory cytokines, including TNF-α, IL-1β, and attenuated overexpression of apoptosis-related genes (CASP3 and Bax) [55]. In vitro studies confirmed that bee venom therapy reduced the production of NO, iNOS, COX-2, PGE2, NF-κB, and pro-inflammatory cytokines (TNF-α, IL-1β, and IL-6) by LPS-induced microglial cells [32,34,56]. Additionally, bee venom increased neuron viability by inhibiting apoptosis through Bax suppression and increasing Bcl-2 gene expression [51].

One of the pilot studies involving 43 patients with idiopathic PD suggests the potential application of bee venom acupuncture as adjunctive therapy for this condition. In this study, patients were divided into three groups: a control group, a group receiving conventional acupuncture, and a group receiving bee venom acupuncture. Patients with PD who received bee venom acupuncture showed significant improvement in overall condition, assessed using both the Unified Parkinson’s Disease Rating Scale (UPDRS) and the Berg Balance Scale [52].

#### 2.3.2. Alzheimer’s Disease

Alzheimer’s disease (AD) is the most common, progressive, incurable neurodegenerative disease leading to patient death, primarily affecting individuals over 65 years old. Its hallmark symptom is cognitive dysfunction, characterized by memory impairments and difficulty in retaining new information. Current research suggests that AD results from a complex interplay of genetic, environmental, and lifestyle factors, leading to irreversible neurodegenerative changes, neuronal death, and loss of intraneuronal connections. The precise causes of the disease remain unclear, but most hypotheses implicate malfunctioning proteins that disrupt normal brain cell function, ultimately leading to their demise. AD pathogenesis is closely linked to the accumulation of β-amyloid and tau proteins in the brain [57]. β-amyloid is derived from amyloid precursor protein, cleaved by β-secretase and γ-secretase enzymes to form β-amyloid molecules. These β-amyloid molecules can aggregate to form toxic soluble oligomers detrimental to neurons. Tau protein, normally involved in transporting nutrients within nerve cells, undergoes conformational changes in AD, forming neurofibrillary tangles that disrupt cellular transport and exert toxic effects [58]. Similar to Parkinson’s disease (PD), treatment for AD is symptomatic. Given the involvement of the cholinergic system in cognitive impairments in AD, the most commonly used drugs are acetylcholinesterase inhibitors [59]. Currently, three ACHE inhibitors—donepezil, galantamine, and rivastigmine—are clinically used for mild to moderate AD [60].

Recent studies indicate the therapeutic potential of bee venom in relation to AD. It has been shown that mice experiencing memory loss induced by LPS exhibited significant inhibition of amyloidogenesis and neuroinflammatory response following bee venom therapy, which involves the inhibition of the NF-κB pathway [61]. Furthermore, subsequent research demonstrated that apamin, a component of bee venom, can improve dendritic morphology in the hippocampus and enhance neuronal excitability and synaptic plasticity in the hippocampus of older rats [62]. Another component of bee venom studied, phospholipase A2, may enhance cognitive functions by deactivating microglia and reducing infiltration of CD4+ T lymphocytes in a mouse model of AD. These findings suggest that bee venom could protect nerve cells from damage by inhibiting inflammation in the nervous system [63].

#### 2.3.3. Amyotrophic Lateral Sclerosis

Amyotrophic lateral sclerosis (ALS) is a severe neurodegenerative disease characterized by the progressive degeneration of motor neurons in the spinal cord’s anterior horns, the brain stem’s cranial nerve nuclei, and the pyramidal pathway neurons. The disease involves a slow, systematic decline in motor function, leading to complete paralysis and ultimately death due to respiratory muscle failure. ALS affects approximately 2–4 per 100,000 individuals, with a higher prevalence among men than women [64].

Transgenic mice treated with bee venom have shown decreased expression levels of microglial markers and phospho-p38 MAPK in the spinal cord and brainstem, along with reduced activity of caspase 3. Additionally, bee venom treatment in animals with ALS improved motor activity and increased survival rates [65]. Administration of melittin to animals exhibiting ALS symptoms reduced the proportion of dead neurons in the spinal cord. Moreover, modifications in α-synuclein (such as phosphorylation or nitration), involved in synaptic formation and neurotransmission regulation, were observed in both the brain stem and spinal cord of transgenic mice. Melittin reduced conformational changes in α-synuclein and restored proteasome activity in the brain stem and spinal cord, contributing to a reduction in nerve inflammation [65].

### 2.4. The Application of Bee Venom in Liver Fibrosis

Liver fibrosis is a progressive condition resulting from chronic exposure to damaging factors such as alcohol, drugs, and inflammatory agents. These factors lead to the replacement of normal liver cells with fibrous tissue, impairing blood flow and consequently disrupting the metabolic functions of the liver, including bile flow obstruction and portal hypertension. Liver damage in fibrosis is irreversible, but the progression of fibrosis can be slowed down [66,67]. The existing research suggests that bee venom may have therapeutic potential for liver fibrosis. Kim et al. demonstrated that bee venom inhibited the progression of liver fibrosis in a mouse model induced by carbon tetrachloride. They observed reduced levels of aspartate aminotransferase (AST) and alanine aminotransferase (ALT) activity in serum, as well as decreased concentrations of pro-inflammatory cytokines (TNF-α, IL-1β) following bee venom administration [68]. Similar results were observed in mice with liver damage induced by acetaminophen, where bee venom, particularly phospholipase A2, exerted protective effects against acetaminophen-induced hepatotoxicity by modulating Treg cells that regulate the immune response [69]. These findings suggest that bee venom may inhibit the inflammatory processes observed in liver fibrosis.

Similar results were obtained in mice with acute liver failure induced by galactosamine and LPS administered intraperitoneally [70]. The animals were randomly divided into four experimental groups: a placebo group treated with physiological saline, a group treated with melittin only, a group subjected to galactosamine and LPS, and a group subjected to galactosamine and LPS with melittin therapy. Liver damage in the studied animals was evaluated biochemically and histologically. The results showed that the expression of TNF-α and IL-1β was increased in the liver cells of the group receiving only galactosamine and LPS, whereas melittin therapy significantly reduced the elevation of these cytokines. Moreover, the group with induced liver failure after melittin therapy exhibited significantly fewer apoptotic cells compared to the group receiving only galactosamine and lipopolysaccharide. Melittin also inhibited the expression of genes encoding caspase 3 and Bax proteins and the release of cytochrome c in hepatocytes. Additionally, melittin prevented the activation of NF-κB induced by galactosamine and LPS [70]. Furthermore, Lee et al. demonstrated that apamin, a component of bee venom, can prevent liver fibrosis by inhibiting the activity of transforming growth factor β (TGF-β). Additionally, this compound prevented morphological changes in hepatocytes and reduced the levels of E-cadherin and vimentin through phosphorylation of Erk1/2, Akt, Smad2/3, and Smad4 [42].

### 2.5. Application of Bee Venom in Atherosclerosis Treatment

Bee venom therapy also includes the use of melittin in the treatment of atherosclerosis. Atherosclerosis is a progressive inflammatory disease of the arteries caused by the accumulation and interaction of white blood cells, remnants of dead cells, cholesterol, and triglycerides within the arterial wall. This complex process involves the presence of monocytes, macrophages, and T lymphocytes. Macrophages secrete pro-inflammatory cytokines, which are key modulators in the development of atherosclerotic plaques [71]. Studies have shown that melittin can positively impact the treatment of atherosclerosis by inhibiting the LPS-induced expression of pro-inflammatory cytokines and adhesion molecules [72,73].

Research using an animal model of atherosclerosis induced by intraperitoneal LPS injections and an atherogenic diet demonstrated that bee venom beneficially affected the reduction in total cholesterol and triglyceride levels in the serum of treated animals. Moreover, animals exposed to bee venom also exhibited decreased levels of pro-inflammatory cytokines, intercellular adhesion molecule-1 (ICAM-1), vascular cell adhesion molecule-1 (VCAM-1), transforming growth factor-β1 (TGF-β1), and fibronectin in the aorta and heart [74]. The therapeutic potential in atherosclerosis has also been confirmed for apamin, constituting 2–3% of the dry weight of bee venom. In studies conducted by Kim et al., mice received intraperitoneal LPS injections to induce atherosclerotic changes and were fed an atherogenic diet. Animals treated with apamin showed reduced expression of genes encoding TNF-α, vascular cell adhesion molecule-1 (VCAM-1), intercellular adhesion molecule-1 (ICAM-1), and NF-κB. Apamin reduced the formation of atherosclerotic lesions, as assessed by hematoxylin staining. Treatment with apamin also decreased serum lipid levels, Ca2+ levels, and TNF-α. Furthermore, apamin significantly attenuated the expression of VCAM-1, ICAM-1, TGF-β1, and fibronectin in the descending aorta of mice with arterial atherosclerosis [75].

### 2.6. Anticancer Effects

Epidemiological studies analyzing the causes of death among beekeepers from 1949 to 1978 revealed significantly lower incidence of cancers, primarily lung cancer, compared to the general population [76]. Havas first described the effect of bee venom on colchicine-induced tumors. Cancer cells are characterized by significant exposure of anionic phospholipids, primarily phosphatidylserine, on the outer surface of their cell membranes [77]. This facilitates easy binding of melittin to cancer cells [78]. Melittin can strongly inhibit the proliferation of cancer cells by suppressing calmodulin, which binds calcium ions. Consequently, melittin inhibits cyclin-dependent kinase activation, chromosome reorganization, DNA synthesis, and cytokinesis. Thus, melittin application resulted in inhibition of in vitro growth and clonogenicity of human (HL-60) and murine (L1210 and L5178Y) leukemia cells [79]. Growth inhibition was also observed in glioma cells following melittin treatment [80]. Furthermore, the combination of bleomycin and melittin increased the mortality of SK-OV-3 ovarian tumor cells, as well as non-tumor cells, including human granulocytes, macrophages, and erythroid progenitor cells [81]. Yang et al. demonstrated that melittin treatment inhibited the growth of head and neck squamous-cell carcinoma (CNE-2 cell line), induced apoptosis in these cells, and reduced the expression of HIF-1α (hypoxia-inducible factor 1α) and VEGF (vascular endothelial growth factor) [82]. Interestingly, melittin exhibits specific cytotoxicity towards cancer cells due to reduced carbohydrate binding sites on their membranes compared to normal bone marrow or splenic cells [83]. Moreover, intraperitoneal injection of melittin significantly reduced the growth of head and neck squamous-cell carcinoma tumors. These findings also suggest that melittin enhances the sensitivity of HNSCC cells to radiation under hypoxic conditions by suppressing HIF-1α expression. Therefore, melittin appears to be a potential sensitizer of cancer cells to radiotherapy [84]. Furthermore, melittin also exhibits anti-angiogenic properties. In vivo studies have shown that melittin can inhibit the growth of BEL-7402 hepatocellular carcinoma cells from mouse xenografts. In this case, the anti-tumor action of melittin may involve reducing the expression levels of VEGF, b-FGF (fibroblast growth factor), and NF-κB, and inhibiting angiogenesis [85].

Melittin is capable of arresting the cell cycle, inhibiting growth, and inducing apoptosis in various cancer cells. Studies using liver cancer and osteosarcoma cell lines confirmed that melittin induces apoptosis by increasing the expression of mitochondrial membrane protein 7A6 and Fas protein [86,87,88]. In leukemia cells, apoptosis activation occurs through increased Bax and caspase 3 activity, and decreased Bcl-2 and inhibitor of apoptosis protein (IAP) family protein activation [89]. In liver cancer, melittin induces apoptosis through caspase 3, caspase 9 cleavage, and poly-ADP-ribose polymerase (PARP). The anti-cancer activity of melittin may involve sensitizing hepatocellular carcinoma cells to apoptosis mediated by TNF-related apoptosis-inducing ligand (TRAIL). Interestingly, melittin combined with TRAIL may be effective in treating TRAIL-resistant cancers [90]. Jo et al. demonstrated that melittin induces apoptosis in ovarian cancer cells by increasing death receptor expression, caspase 3 and caspase 8 activity, and Bax protein while reducing Bcl-2 expression through enhanced JAK2 and STAT3 kinase phosphorylation [91]. Both in vitro and in vivo studies confirmed that melittin could be a potential therapeutic agent for hepatocellular carcinoma. Melittin can inhibit the metastasis of cancer cells by reducing their motility and migration through suppression of the Rac1-dependent pathway [46].

In studies using phorbol myristate acetate (PMA) as an activator of matrix metalloproteinase 9 expression, melittin inhibited invasion of kidney cancer cells. Matrix metalloproteinase 9 plays a crucial role in the formation of tumor metastases. Melittin suppressed the expression of this protein by blocking activator protein 1 (AP-1) and NF-κB activation [92]. Furthermore, melittin may also inhibit the progression of cervical cancer. In vitro, melittin specifically suppressed epidermal growth factor (EGF)-induced VEGF release, thereby inhibiting HIF-1α and neovascularization [93].

An interesting area of research involves the use of bee venom, and specifically melittin, in combination with other chemotherapeutic agents. Duffy et al. observed that both bee venom and melittin exhibited cytotoxic effects in cell lines of particularly aggressive breast cancer, specifically triple-negative breast cancer (TNBC), which lacks expression of estrogen, progesterone, and HER2 receptors, as well as in a HER2-enriched TNBC cell line. The mechanism of action involved the phosphorylation of EGFR and HER2 receptors in the plasma membrane, leading to reduced receptor activation. Additionally, the study found that melittin sensitized cells to docetaxel, a widely used classic cytostatic agent. The combination of melittin and docetaxel resulted in significant tumor growth inhibition following allogeneic transplantation [94]. Melittin also exhibits a synergistic antitumor effect in breast cancer cells when combined with tamoxifen, hesperidin, and piperine [95]. In turn, Mansour et al. demonstrated that melittin exhibits a synergistic antitumor effect with sorafenib, which has suboptimal efficacy in treating hepatocellular carcinoma, in HepG2 cell lines [96]. Similarly, Nusair et al. reported that melittin, in combination with sorafenib, also showed synergistic activity in HepG2 cells by inducing both apoptosis and autophagy mechanisms of cell death [97]. In lung cancer and squamous cell carcinoma lines, melittin sensitized cells to cisplatin and 5-fluorouracil, respectively, by affecting cell cycle arrest and significantly reducing tumor size [98,99].

In vitro research into the antitumor effects of bee venom and melittin—both alone and in combination with standard chemotherapeutic agents—has demonstrated promising results. These studies suggest that melittin could serve as a valuable adjunct to existing cancer therapies, potentially addressing resistance to conventional treatments. However, for these findings to be translated into clinical practice, further in vivo studies are necessary. Such studies are crucial for evaluating the safety, efficacy, and optimal administration of these compounds in living organisms. Successful in vivo research could facilitate clinical trials and potentially establish bee venom and melittin as viable therapeutic options, particularly for cancers resistant to traditional therapies. Therefore, while in vitro results are encouraging, comprehensive in vivo investigations are essential to advance these potential treatments from the laboratory to clinical application.

## 3. Toxicity of Bee Venom

Despite numerous studies demonstrating the therapeutic effects of bee venom, its use remains highly restricted due to safety concerns. It can cause a range of adverse effects, most of which are unpredictable. A meta-analysis of therapeutic studies on bee venom showed that out of 145 studies, 58 reported adverse effects. The most common side effects observed were systemic immune reactions, local reactions, allergies, and skin problems [100,101,102,103]. These adverse reactions are often due to hypersensitivity, frequency, and concentration of administered bee venom [101,104]. An unpredictable reaction to bee venom therapy was noted in a 55-year-old woman from Spain. The woman tolerated apitherapy treatments well for two years. However, a subsequent treatment resulted in a severe anaphylactic reaction, including difficulty breathing, wheezing, and loss of consciousness, and ultimately led to her death [105]. In vivo studies on rodents determined the lethal dose of bee venom to be 13.19 mg/kg. However, the case of the Spanish woman highlights that the dose received from a bee sting, approximately 50–140 µg, can also be lethal for an individual [11,105].

During a sting, a bee injects up to 0.3 mg of venom [106]. The main allergens in bee venom are phospholipase A2, apamin, hyaluronidase, acid phosphatase, melittin, and protease inhibitor, which disrupt cell membranes, degrade hyaluronic acid, destroy extracellular matrix, and facilitate the spread of venom in the body [107,108]. Previous studies indicate that these allergens can trigger allergic reactions in up to 50% of the population, which can include skin changes and affect the gastrointestinal, cardiovascular, and respiratory systems, and in severe cases, can lead to death [105,109].

In cases where a large dose of bee venom is introduced, such as during simultaneous stings by several dozen bees or more, symptoms of a toxic reaction may emerge as a result of the direct cytotoxic effects of the venom [3]. Garaj-Vrhovac and Gajski demonstrated that high doses of bee venom (100 μg/mL) exhibited genotoxic effects in human lymphocytes [110]. The venom components—phospholipase A2 and melittin—induce hemolysis, liquefactive necrosis of striated muscle, and thrombocytopenia. Melittin is classified as a lytic peptide that disrupts phospholipid membranes, leading to hemolysis through the induction of conformational alterations in the lipid bilayer, resulting in the formation of pore-like structures. Moreover, these sites are preferentially targeted by phospholipase A2 (PLA2), which leads to a synergistic effect between these proteins, further enhancing membrane degradation and increasing the cytotoxicity [111].

The toxic reaction can be immediate or delayed. Most symptoms of an immediate reaction overlap with those of an allergic reaction, with severity depending on the dose received and individual tolerance. Common symptoms of an immediate toxic reaction include fatigue, nausea, vomiting, diarrhea, hypotension, and shock [3]. Laboratory tests typically reveal signs of hemolysis, liver dysfunction (elevated liver enzymes: ALT, AST), thrombocytopenia, elevated creatine kinase (CK) levels, and increased concentrations of cardiac enzymes (e.g., troponins). Acute kidney failure may occur due to rhabdomyolysis, hemolysis, or episodes of hypotension [112]. In contrast, delayed toxic reactions are rare but can manifest up to 24 h after the initial exposure. Initially, the patient, aside from pain caused by the stings, may not experience any other symptoms, and laboratory test results may appear normal. Only after several hours do signs of multi-organ dysfunction begin to develop [113]. Characteristic symptoms of a delayed toxic response include hemolysis, hemoglobinuria, rhabdomyolysis, and thrombocytopenia. Additional possible symptoms include tubular necrosis, disseminated intravascular coagulation (DIC), and damage to the liver and central nervous system, most commonly presenting as visual impairment and psychotic symptoms [114]. Early intervention and intensive therapy, including aggressive intravenous hydration, blood transfusions, and, if necessary, dialysis, can prevent the progression of these permanent complications [3,114].

## 4. Isolation, Purification, and Allergy Components of Bee Venom

The predominant method currently employed for the extraction of bee venom is electrical stimulation, which, while regarded as safe for *Apis mellifera*, involves considerable technical complexity. As a result, only a limited number of specialists are engaged in optimizing this technique. Venom extraction is facilitated by devices designed in the form of standard-sized frames, which can be readily inserted into a hive. These devices feature a glass plate housed within a specialized enclosure that emits an electrical current of extremely low intensity. This current is imperceptible to humans but sufficiently stimulates the bees, triggering a defensive response. The bees respond by stinging the glass plate, depositing venom that rapidly solidifies and is subsequently collected by scraping. This technique is advantageous as it does not result in the loss of the stinger. Empirical studies have demonstrated that this extraction method does not adversely affect the bees’ productivity, health, or overwintering survival rates. The glass plate is only temporarily installed within the hive, ensuring that venom is not harvested continuously. Despite the overall safety of the method, the installation of the device has been observed to induce a moderate stress response in the bees, as indicated by increased activity levels. To mitigate potential disruptions to the colony, venom collection is timed during periods of reduced foraging activity to minimize interference with the colony’s natural rhythm. Furthermore, considering the temporary depletion of the bee’s venom reserves following a sting, apitoxin is harvested at a strictly controlled frequency, determined by factors such as the bee species and the specific management practices employed within the apiary [115].

However, to enhance the safety of bee venom use, researchers suggest removing allergens such as phospholipase A2, hyaluronidase, apamin, and acid phosphatase. Lee et al. proposed a method for purifying bee venom that allows the removal of phospholipase A2 and apamin [116]. High-performance liquid chromatography (HPLC) was performed using a C18-5E YMC (5 µm, 4.6 × 150 mm) column with a Waters Alliance UV detector. Melittin is relatively easy to reduce or degrade under physical and chemical conditions such as heat, acids, or bases. Therefore, 50% aqueous ethanol was used as the solvent in this study, as it does not affect melittin content during purification and analysis of raw bee venom, and additionally reduces apamin content in the solution. In this method, the raw bee venom sample was diluted to 10% and purified on an open column with a step gradient (ODS-A, 120 Å and 150 mesh), eluted using 0–80% ethanol. To isolate and purify the active component of raw bee venom (10 g/mL), the raw venom was divided into 13 fractions. According to the respective HPLC profiles, fractions 1–5 (~10% ethanol layer) contained no compounds. Apamin first appeared in fraction 6, and fraction 7 contained both apamin and PLA2. Melittin was eluted in fraction 10, but it was mixed with PLA2. Pure melittin was obtained in the 70–80% ethanol layer. The composition of each fraction obtained by open-column chromatography was determined by HPLC analysis using apamin, PLA2, and melittin as standard compounds. Using standard ingredients, apamin was detected after 12 min, PLA2 in two peaks after 18 and 19 min, and melittin after 27 min. Melittin was detected in fraction 11 (70% ethanol layer), and the peak area (%) was approximately 98%. The standard purity of melittin was 99.4%, and the melittin content in purified bee venom was 99% higher than the commercial standard. The total yield of melittin was 63%, and its purity after separation and purification was approximately 92–99%. Purified bee venom was concentrated and lyophilized (concentrated under reduced pressure) to produce a powder. The melittin content in purified bee venom was determined by HPLC [116].

Han et al. patented a method for purifying bee venom using ultrafiltration in the range of 0.57–1 g of apitoxins/L. The main advantage of this process is the use of mild and modular conditions, which are crucial for large-scale bee venom production. Additionally, during ultrafiltration on a commercially available 10 kDa membrane, it is possible to remove phospholipase A2, which, like apamin, has strong allergenic effects [117].

## 5. Future Perspectives

The most potent cytolytic peptide derived from bee venom is melittin, whose biological properties predispose it for therapeutic use in various diseases. However, a significant limitation arises from observed adverse effects in vivo, primarily associated with hepatotoxicity and hemolysis. These adverse effects pose significant risks and can limit the peptide’s therapeutic window, making it less feasible for widespread clinical use [118]. Additionally, melittin’s inherent properties present further obstacles. Its low stability in biological environments means that it may degrade or lose effectiveness before reaching its target. Poor tissue penetration is another critical issue, as it hampers the peptide’s ability to reach and act on deep or internal tissues effectively. These factors collectively reduce the peptide’s therapeutic efficacy and increase the risk of adverse reactions. To overcome these challenges, researchers are exploring various strategies to enhance melittin’s therapeutic potential. One approach involves the development of conjugates, where melittin is chemically linked to other molecules to improve its stability, target specificity, or therapeutic properties. For instance, conjugation with targeting ligands can help direct melittin to specific cells or tissues, reducing off-target effects and enhancing its efficacy. Nanotechnology offers another promising avenue for improving melittin’s functionality. Encapsulation of melittin in nanostructures such as liposomes, nanoparticles, or hydrogels can enhance its stability, control its release, and improve its tissue penetration. These nanocarriers can protect melittin from degradation, facilitate its delivery to target sites, and reduce systemic toxicity by minimizing exposure to non-target tissues [119].

As part of these efforts, a conjugate was developed containing recombinant immunotoxin melittin and a single-chain antibody with a modified fragment of the asialoglycoprotein receptor (ASGPR), contributing to melittin’s specific cytotoxicity against hepatocellular carcinoma cells. This modification retained hemolytic activity and exhibited cytolytic activity against HepG2 cells at a concentration of 1.5 µg/mL, with no observed erythrocyte lysis. Treatment of HepG2 cells with the conjugate resulted in 68% cell death, as measured by trypan blue staining [120].

To reduce the number of human breast cancer cells overexpressing HER2, a complex was developed comprising pegylated immunoliposomes as carriers, trastuzumab antibody for targeted transport, and melittin as the active substance. Using a panel of human breast cancer cells (MCF7/HER2, SKBr3, JIMT-1, MCF7—ranked by HER2 expression levels from highest to lowest), it was demonstrated that the investigated immunoliposomes reduced the viability of cancer cells in a dose-dependent manner correlated with HER2 expression levels [121]. A similar approach using pegylated immunoliposomes was developed against liver cancer cells. Due to the hemolytic activity of bee venom, stabilized liposomal bee venom was developed using soybean phosphatidylcholine, cholesterol, and pegylated cholesterol. Studies in an in vitro model involving human liver cancer SMMC-7721 cells demonstrated greater effectiveness of the developed complexes compared to non-targeted liposomes [122]. However, these solutions may have limitations in therapeutic application due to the potential release of melittin into blood vessels during transport [123].

Therefore, a carrier was developed using a perfluorocarbon nanoemulsion containing melittin in an external lipid monolayer [124]. This carrier demonstrated favorable in vivo pharmacokinetics, accumulating melittin in mouse tumors and causing a significant reduction in tumor growth without observable systemic toxicity [125,126]. Another melittin nanoparticle with a very small diameter (<40 nm), known as α-melittin-NP, was successfully tested in vivo with minimal side effects [127,128]. This nanoparticle contains 1,2-dimyristoyl-sn-glycero-3-phosphatidylcholine surrounded by a hybrid peptide formed by peptide D-4F and melittin via a GSG linker, where peptide D-4F mimics high-density lipoproteins (HDLs) [129].

Overall, while melittin shows considerable promise as a therapeutic agent, addressing its current limitations through innovative modifications and delivery systems is crucial for realizing its full potential in clinical applications. Continued research and development in these areas are essential for optimizing melittin’s therapeutic efficacy and safety profile.

## 6. Conclusions

Apitoxinotherapy, or bee venom therapy (BVT), represents an innovative approach to treating various diseases, with notable therapeutic potential. The chemical constituents of bee venom, especially melittin, apamin, phospholipase A2, and bioamines like histamine and catecholamines, contribute to a wide array of biological activities exhibited by BVT. These include anti-inflammatory, analgesic, antimicrobial, antiviral, neuroprotective, antiarthritic, and anticancer properties. Despite the evident therapeutic benefits of BVT, a critical concern is the immunogenicity of bee venom, which presents a challenge to its safe application. Further research aimed at reducing the presence of highly allergenic proteins is necessary to enhance therapy safety while preserving its therapeutic efficacy.

In conclusion, apitoxin therapy holds significant promise as a complementary treatment method, yet additional research is essential to optimize its safety and effectiveness. These efforts have the potential to expand the utilization of bee venom in medical practice, offering new therapeutic options for numerous patients.

## Figures and Tables

**Table 1:** Types of bee venom therapy.

Type of Therapy	Description	References
Apipuncture	Purified and diluted bee venom injected into specific acupuncture points to optimize therapeutic effects	[2]
Bee stings	Bee sting administered at a specific site; this method carries a high risk of fatal anaphylactic reactions	[3]
Injection therapy	Injection of bee venom at a precisely defined location	[7]
Topical application	Direct application of bee venom ointments or creams to the skin for localized treatment	[5]
Electrotherapy with bee venom	Combination of bee venom with electrical stimulation to enhance therapeutic effects	[9]

## Data Availability

Data sharing is not applicable.
